# The complete mitochondrial genome of *Corythucha marmorata* (Hemiptera: Tingidae)

**DOI:** 10.1080/23802359.2017.1407712

**Published:** 2017-11-27

**Authors:** Aili Lin, Xincheng Zhao, Xinxin Li, Nan Song

**Affiliations:** College of Plant Protection, Henan Agricultural University, Zhengzhou, People’s Republic of China

**Keywords:** Mitochondrial genome, *Corythucha marmorata*, phylogenetic analysis

## Abstract

The complete mitochondrial genome (mitogenome) of *Corythucha marmorata* has been sequenced and annotated. The entire mitogenome is a typical circle double-stranded DNA molecule of 15,635b p and consisted of 37 genes and a control region in the typical invertebrate mitochondrial gene arrangement. Phylogenetic analysis recovered the momophyly of Gerromorpha, Enicocephalomorpha and Cimicomorpha.

The lace bug, *Corythucha marmorata*, belonging to the family Tingidae in the order Hemiptera. The genus *Corythucha* which does harm to trees and plants distributed originally in the Nearctic Region and Neotropical region, included 49 species (Mutun et al. 2009). To date, six mitogenomes have been sequenced from the family Tingidae, only one mitogenome come from the genus *Corythucha* (Yang et al. 2013). In this study, *C. marmorata* was collected from city of Zhengzhou, China (the geospatial coordinates: 113.635°E, 34.723°N), and the primary specimen can be obtained by the Entomological Museum of Henan Agricultural University (voucher no. MT-Zz15100216). Genomic DNA was isolated from muscular tissue preserved in the absolute ethyl alcohol using the TIANamp Micro DNA Kit (TIANGEN BIOTECH CO., LTD, Beijing, China) following the protocol of the manufacturer and then subjected to build up a genomic pool with the Genomic DNA of other insects, pair-end sequencing (2 × 150) by next-generation sequencing (NGS) method from the Illumina HiSeq 2500 (Illumina, San Diego, CA). The raw reads were de novo assembled by the SOAPdenovo software (Zhao et al. 2011), with an average 358.98 × coverage. We identified the complete mitogenome of *C. marmorata* from an assembled contig of 15,752 bp by the *cox1* bait sequence, through blasting against the assembled contigs by BioEdit7.0.9.0 (Hall 1999). The GenBank accession number of *C. marmorata* is MG479390.

The complete mitogenome of *C. marmorata* is a circle double-stranded molecule of 15,635 bp, including 13 protein-coding genes, two rRNA genes, 22 tRNA genes and a control region. The gene arrangement is consistent with most arthropod mitogenomes (Wolstenholme 1992). The contents of A + T and G + C are 77.50% and 22.50%, respectively. All protein-coding genes start with the typical codon ATN, but only *cox2*, *cox3*, *nad5* and *nad4l* stop with incomplete terminal codons T/TA rather than typical stop codon TAA/TAG. The 22 tRNA genes have the conventional cloverleaf structure except for trnS1 which lacks the dihydrouridine (DHU) arm. The *lrRNA* locates between *trnL1* and *trnV* genes, and the *srRNA* between *trnV* gene and control region. The length of the *lrRNA* and *srRNA* genes is 1221 bp and 754 bp, respectively.

The length of full control region is 1220 bp, with the A + T content of 68.03%. The length of this region of *C. marmorata* is longer than *C. ciliate* (Yang et al. 2013). There are three tandem repeat units detected in the control region, of which the longest one includes 92 bases.

Phylogenetic analysis shows that Gerromorpha and Enicocephalomorpha are monophyletic groups (Yang et al. 2013; Figure 1). The monophyly of Cimicomorpha supported is consistent with previous studies (Schuh 1979; Zrzavy 1992; Mahner 1993; Scherbakov and Popov 2002; Yang 2002; Xie et al. 2008; Weirauch and Schuh 2011), but incongruent with Yang et al. (2013). Within Cimicomorpha, Tingidae and Reduviidae are monophyletic, but Miridae is paraphyletic (Li et al. 2011, Li, Liu, Shi et al. 2012; Li, Liu, Cao, et al. 2012; Yang et al. 2013). Tingidae is a sister group to the rest of Cimicomorpha.

**Figure 1.**
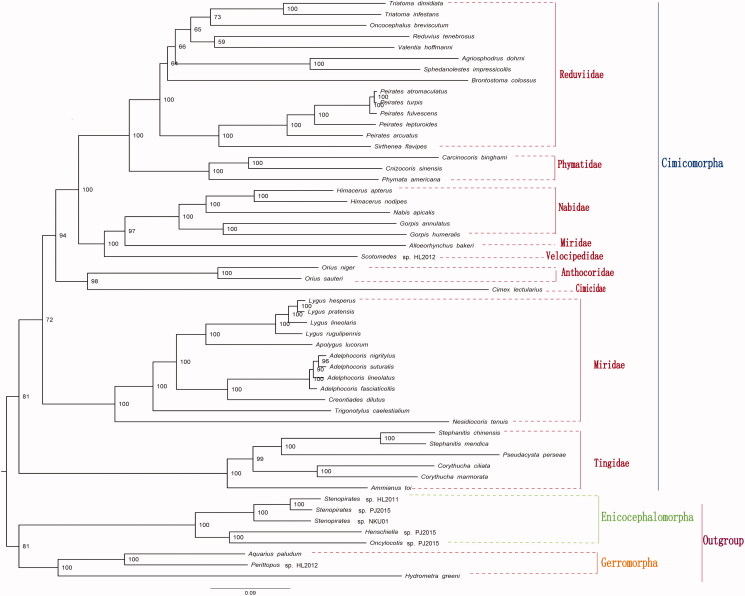
Maximum-likelihood tree of complete mitogenomes of *C. marmorata* and 52 other species from GenBank. The maximum-likelihood analysis was reconstructed by concatenated nucleotide sequences of 13 mitochondrial protein genes (10,968 bp) using IQ-TREE (Nguyen et al. 2015). Numbers alongside nodes refer to bootstrap support values. All 53 species accession numbers are listed as below: *Trigonotylus caelestialium* (KJ170899), *Adelphocoris fasciaticollis* (KU234536), *A. lineolatus* (KJ020286), *A. nigritylus* (KJ020287), *A. suturalis* (KJ020288), *Alloeorhynchus bakeri* (NC_016432), *Apolygus lucorum* (NC_023083), *Creontiades dilutus* (NC_030257), *Lygus hesperus* (KF679984), *L. rugulipennis* (KJ170898), *L. lineolaris* (NC_021975), *L. pratensis* (KU234540), *Nesidiocoris tenuis* (NC_022677), *Pseudacysta perseae* (NC_025299), *Stephanitis chinensis* (MF498769), *S. mendica* (JQ739184), *Ammianus toi* (JQ739178), *C. marmorata* (), *C. ciliata* (KC756280), *Gorpis annulatus* (NC_019595), *G. humeralis* (NC_019593), *Himacerus apterus* (JF927831), *H. nodipes* (JF927832), *Nabis apicalis* (NC_019594), *Orius niger* (NC_012429), *O. sauteri* (NC_024583), *Scotomedes* sp.HL-2012 (JQ743677), *Cimex lectularius* (KU350871), *Reduvius tenebrosus* (NC_035756), *Triatoma infestans* (NC_035547), *Agriosphodrus dohrni* (NC_015842), *Brontostoma colossus* (NC_024745), *Peirates lepturoides* (NC_026672), *P. turpis* (NC_026671), *P. atromaculatus* (NC_026670), *P. fulvescens* (NC_026669), *P. arcuatus* (NC_024264), *Sirthenea flavipes* (NC_020143), *Oncocephalus breviscutum* (NC_022816), *Triatoma dimidiata* (NC_002609), *Valentia hoffmanni* (NC_012823), *Sphedanolestes impressicollis* (KC887536), *Cnizocoris sinensis* (NC_036013), *Carcinocoris binghami* (NC_036012), *Phymata americana* (NC_036011), *Perittopus* sp. HL-2012 (JQ910988), *Hydrometra greeni* (NC_012842), *Aquarius paludum* (NC_012841), *Stenopirates* sp. HL-2011 (NC_016017), *Stenopirates* sp. PJ-2015 (KP406518), *Oncylocotis* sp. PJ-2015 (KP406517), *Henschiella* sp. PJ-2015 (KP406516), *Stenopirates* sp. NKU01 (JN989543).
